# A lipid-related transcriptomic signature for distinguishing Kawasaki disease from febrile infections: a Mendelian randomization and machine-learning study

**DOI:** 10.3389/fimmu.2026.1851332

**Published:** 2026-09-30

**Authors:** Sibao Wang, Gang Luo, Zhixian Ji, Guoxiang Zhou, Silin Pan

**Affiliations:** Heart Center, Women and Children’s Hospital, Qingdao University, Qingdao, China

**Keywords:** differential diagnosis, endothelial activation, Kawasaki disease, lipid metabolism, machine learning, Mendelian randomization

## Abstract

**Background:**

Kawasaki disease (KD) diagnosis remains challenging because no pathognomonic test exists and the clinical presentation overlaps extensively with common childhood infections. Acute KD is accompanied by reproducible lipid perturbations, yet whether these changes are causally linked to the innate immune-driven vascular damage of KD vasculitis is unestablished. We aimed to derive a lipid-related transcriptomic signature for distinguishing KD from febrile infections and to characterize its relationship with innate immune remodeling.

**Methods:**

Bidirectional two-sample Mendelian randomization (MR) screened 422 candidate exposures (419 lipid/metabolic traits and three immune-cell phenotypes) against two independent KD GWAS cohorts, with multiplicity correction. Differentially expressed genes from a training cohort comparing KD with rigorously classified febrile controls were intersected with a broad lipid-metabolism-related GeneCards set, yielding 459 candidates. The 459 candidates were evaluated across 130 machine-learning combinations; a full-training LASSO fit at lambda.1se yielded a 12-gene candidate panel, and the saved penalized model was applied unchanged to an independent cohort without batch correction. The immune microenvironment was estimated using CIBERSORT, acute-to-convalescent expression changes were tracked longitudinally, and selected markers were assessed in TNF-α-stimulated HUVECs.

**Results:**

No exposure survived correction for the 422 tests performed (minimum Benjamini–Hochberg q = 0.979). All nine discovery-stage nominal hits were evaluated in a second KD GWAS; eight had directionally concordant point estimates, but none reached an unadjusted P < 0.05 in the second cohort. The frozen penalized model achieved an external AUC of 0.851 (95% CI 0.768–0.934) in GSE68004 without cross-cohort batch correction. CIBERSORT indicated a higher estimated neutrophil fraction in KD, concordant with a mature-neutrophil marker score from the same matrix. CETP, KREMEN1, and CCL23 were lower in convalescent samples after IVIG (all FDR < 0.0001) and were induced in TNF-α-treated HUVECs, together with VCAM-1, ICAM-1, and IL-8.

**Conclusions:**

The frozen penalized model showed moderate discrimination between KD and febrile controls, but the selected 12-gene set represents one candidate realization of a partition-sensitive modeling pipeline. KREMEN1 warrants separate biological study on the basis of marginal, longitudinal, and immune-association findings rather than its selection stability. Recalibration and prospective validation are required before clinical use. The HUVEC findings are transcriptional and hypothesis-generating.

## 1 Introduction

Early diagnosis of Kawasaki disease remains difficult. High-dose IVIG, when administered early, substantially curtails the risk of coronary artery aneurysms (CAA), but the treatment window is narrow and no pathognomonic laboratory marker has yet been identified (1, 2). Diagnosis relies on clinical features that overlap with those of adenovirus infection, group A Streptococcus infection, and other common febrile illnesses in children (3). Missed or delayed recognition therefore persists as a significant source of preventable cardiovascular harm among febrile children (4). This diagnostic gap has stimulated a growing body of transcriptomic classifier studies, many reporting AUC values in excess of 0.95 (5). However, many published classifiers were trained against healthy controls. Classifiers trained on that contrast tend to capture a generic inflammatory signal shared with numerous acute conditions rather than biology specific to KD. Performance accordingly deteriorates once the benchmark shifts to another acutely febrile illness, which is precisely the distinction that matters at the bedside (1, 5).

Lipid metabolism may offer a more disease-specific entry point. Transient HDL depression and pronounced VLDL cholesterol remodeling have been reported consistently in acute KD (6–8), yet whether these perturbations participate causally in the endothelial injury that defines KD vasculitis or merely reflect the metabolic fallout of systemic inflammation remains unresolved. Endothelial activation and innate immune chemotaxis lie at the mechanistic heart of coronary arteritis in KD (9, 10); examining genetic and transcriptomic evidence in parallel may help contextualize lipid-related biomarker candidates without implying molecular mediation.

The innate immune dimension of KD pathogenesis is central to this study. Pathological studies of coronary arteries in fatal KD cases have demonstrated that neutrophil infiltration precedes and exceeds that of monocytes and lymphocytes, suggesting that neutrophils are involved early in vascular wall injury (11). Circulating neutrophils in acute KD exhibit prolonged survival, enhanced respiratory burst, and a marked propensity for spontaneous formation of neutrophil extracellular traps (NETs)—web-like chromatin structures decorated with cytotoxic granule proteins that can directly injure the vascular endothelium (12, 13). How this neutrophil-dominated immune landscape intersects with the lipid-metabolic abnormalities of acute KD has received little systematic attention.

Mendelian randomization uses genetic variants as instruments to evaluate associations that may be less susceptible to confounding and reverse causation than conventional observational analyses (14). Here, MR was used as a separate hypothesis-generating analysis of genetically proxied exposure associations with KD; it did not define or narrow the transcriptomic candidate set. Training the transcriptomic model against febrile controls was intended to match the clinically relevant comparison—KD versus febrile illness, not KD versus health (15).

Here we report a framework comprising parallel genetic and transcriptomic analyses followed by immune and longitudinal characterization. Bidirectional MR screened 422 candidate exposures, and all nine discovery-stage nominal hits were subsequently evaluated in a second KD GWAS. Independently, KD-versus-FC differentially expressed genes were intersected with a broad lipid-metabolism-related GeneCards set, and the resulting candidates entered a machine-learning pipeline in which one full-training LASSO fit yielded a 12-gene candidate panel. The panel was evaluated in an independent cohort without cross-cohort batch correction. We then examined acute-to-convalescent expression changes and estimated peripheral-blood immune-cell proportions. Finally, the transcriptional responses of CETP, KREMEN1, and CCL23 were assessed in TNF-α-treated HUVECs.

## 2 Materials and methods

### 2.1 Overall study design

The study was organized in four stages (**Figure 1**). Bidirectional two-sample MR screened 422 candidate exposures for genetically proxied associations with KD. Independently, KD-versus-FC DEGs were intersected with a broad lipid-metabolism-related GeneCards set, and the resulting candidates were used to derive a parsimonious diagnostic panel. Exploratory transcriptional responses of CETP, KREMEN1, and CCL23 were assessed in a TNF-α-stimulated HUVEC inflammatory model.

**Figure 1:**
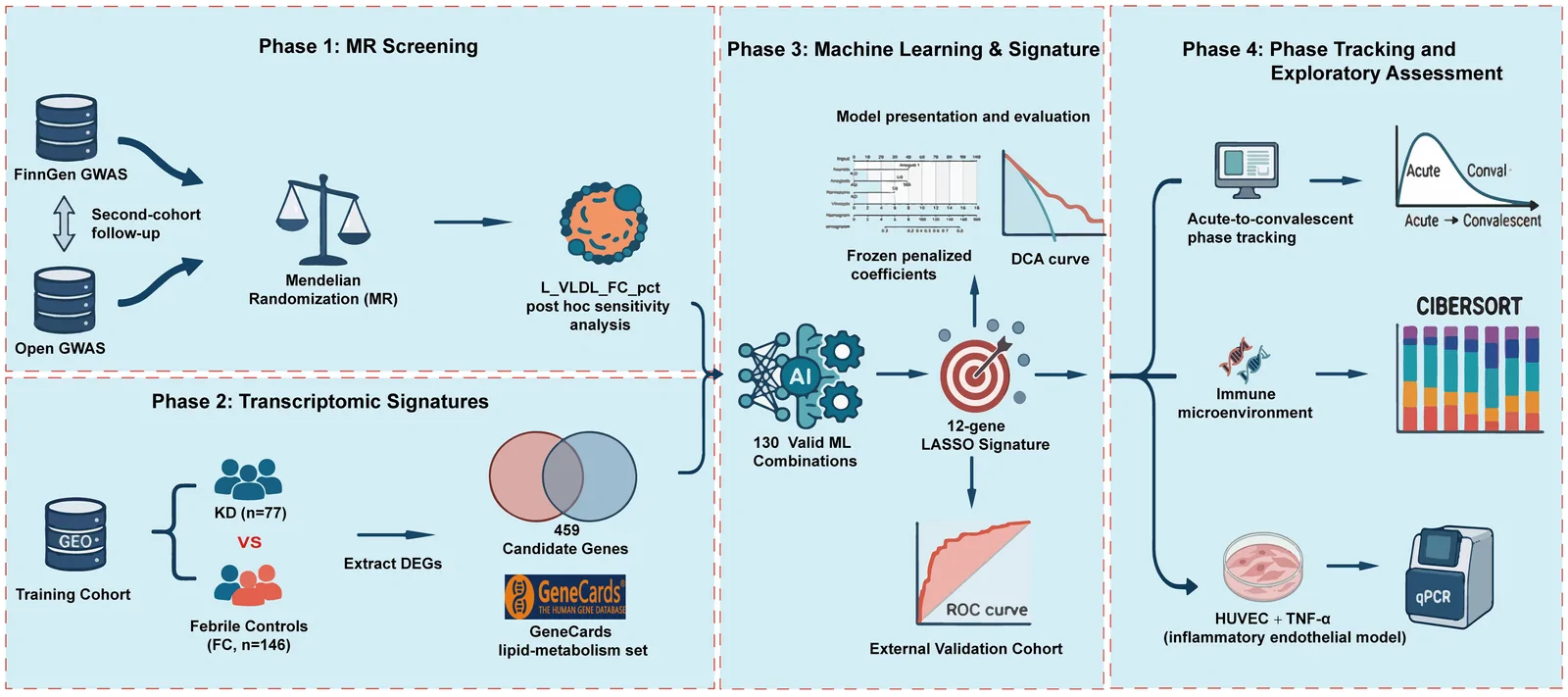
Overall study design and analytical workflow. The study comprises four phases. Phase 1: bidirectional MR screened 422 candidate exposures for association with KD susceptibility, and all nine discovery-stage nominal hits were evaluated in a second KD GWAS; eight point estimates were directionally concordant, but none reached an unadjusted P < 0.05 in the second cohort. Phase 2: independently, DEGs distinguishing KD from strictly classified febrile controls were intersected with a broad lipid-metabolism-related GeneCards set, yielding 459 candidates. Phase 3: one 12-gene candidate realization was obtained from the full-training LASSO fit and evaluated as a frozen penalized model. Phase 4: the signature was examined through acute-to-convalescent expression changes, immune-deconvolution analyses, and exploratory transcriptional assessment in TNF-α-stimulated HUVECs.

### 2.2 Two-sample bidirectional Mendelian randomization

#### 2.2.1 GWAS data sources

Two independent GWAS datasets provided KD susceptibility data: the FinnGen consortium (R12 release, European ancestry) (16) and the OpenGWAS database (ID: ebi-a-GCST90014243, European ancestry). A total of 422 candidate exposures were retrieved from four OpenGWAS batches (met-a through met-d), which differed in source study, measurement platform, and sample size. All four batches were derived from European-ancestry participants. The screened set comprised 419 lipid/metabolic traits and three immune-cell phenotypes from the met-b batch. The met-c batch comprised 114 Nightingale NMR metabolite traits from a published metabolite GWAS (17); dataset-level metadata for all four batches are provided in **Supplementary Table 10**. The three met-b phenotypes were included in the original screening list because of exposure-list misclassification; none approached significance, and excluding them changes no reported result. Because all 422 exposures were screened, all were retained in the denominator for multiple-testing correction. L_VLDL_FC_pct, the trait examined in *post hoc* sensitivity analyses, derives from the met-d batch (UK Biobank Nightingale panel); UK Biobank and FinnGen are separate national cohorts, and no participant overlap is expected for this estimate.

#### 2.2.2 Instrumental variable selection and MR analysis

Genome-wide significant single nucleotide polymorphisms (SNPs) (P < 5 × 10^−8^) for each exposure served as instrumental variables. Independence was enforced through linkage disequilibrium clumping (r² < 0.001, window = 10,000 kb) against the 1000 Genomes European panel; SNPs with F-statistics below 10 were removed. The inverse-variance weighted (IVW) method served as the primary estimator, with consistency checked by MR-Egger regression, weighted median, and mode-based estimators. Heterogeneity was assessed using Cochran’s Q test, directional pleiotropy by the MR-Egger intercept, and the influence of individual SNPs through leave-one-out analysis. All MR analyses were conducted with the TwoSampleMR package (version 0.5.8) in R (18, 19). For the reverse direction, KD liability (FinnGen R12) served as the exposure and L_VLDL_FC_pct as the outcome. Because no locus reached genome-wide significance, a relaxed threshold of P < 5 × 10^−6^ was applied, and the resulting variants were assessed for allele frequency, genomic annotation, and instrument plausibility before harmonization (**Supplementary Table 12**).

Because 422 exposures were screened, Bonferroni-corrected P values (α = 0.05/422 = 1.18 × 10^−4^) and Benjamini–Hochberg q values were calculated for every trait. All nine exposures with an unadjusted FinnGen P < 0.05 were subsequently evaluated in the second KD GWAS (ebi-a-GCST90014243) using the same exposure-instrument criteria. Exact outcome variants were queried first; for unavailable variants, proxies with LD r² ≥ 0.80 were accepted. Raw target-to-proxy mappings were retained and the actual outcome variants were audited before duplicate-proxy resolution, preventing the same outcome proxy from being counted more than once within an exposure. Harmonization used action 2, and inverse-variance weighting was the primary estimator when at least two instruments were available. Because this follow-up was not prespecified, direction concordance with an unadjusted second-cohort P < 0.05 was defined as a directionally concordant nominal association rather than formal replication. Bonferroni- and Benjamini–Hochberg–adjusted P values across the nine follow-up tests were also calculated. Outcome GWAS characteristics and sample-overlap assessment are provided in **Supplementary Table 11**, and complete second-cohort results are provided in **Supplementary Table 18**. L_VLDL_FC_pct was retained for *post hoc* exploratory sensitivity analyses as a representative VLDL-composition trait with a comparatively large instrument set. It was not selected on the basis of the smallest discovery-stage or second-cohort P value. Additional sensitivity analyses included MR-PRESSO, Steiger directionality testing, leave-one-locus-out analysis, and multivariable MR adjusting for LDL cholesterol, HDL cholesterol, triglycerides, apolipoprotein B, and apolipoprotein A1; conditional F-statistics are reported in **Supplementary Table 12**.

### 2.3 Transcriptomic data acquisition and processing

Because the clinically relevant question is whether a febrile child has KD—not whether a child is sick or healthy—we deliberately selected Gene Expression Omnibus (GEO) datasets that included well-characterized FC (**Supplementary Table 1**).

The training cohort (GSE73461) initially comprised 78 patients with KD and 146 febrile controls, restricted to definite bacterial (DB, n = 52) and definite viral (DV, n = 94) infections; indeterminate cases and systemic autoimmune conditions were excluded (15). One KD sample (GSM1894671) contained no expression measurements and was excluded before analysis, leaving 77 KD and 146 febrile-control samples. The independent validation cohort (GSE68004) included 89 patients with KD and 36 febrile controls with adenovirus or group A Streptococcus infection, profiled on the same Illumina HumanHT-12 V4.0 platform as the training cohort (20). The dynamic tracking cohort (GSE63881) included 171 patients with KD sampled during the acute phase and, for 170 of them, again during convalescence; one patient lacked a convalescent sample and was excluded from paired analyses (21).

Probe identifiers were mapped to official gene symbols using the corresponding platform annotation. For genes represented by multiple probes, the maximum expression value across mapped probes was retained separately for each sample. The DEG–GeneCards intersection was used only to define a lipid-metabolism-enriched search space; it does not establish that the selected genes are downstream effectors of L_VLDL_FC_pct or genetically anchored to the MR association. DEGs were identified with the limma package (22) at |log_2_FC| > 0.585 and adjusted P < 0.05. The 1,358 DEGs were intersected with 9,603 lipid-metabolism-related genes from GeneCards (relevance score > 1.0) (23), yielding 459 candidates. A permutation test (10,000 draws) assessed whether signature genes were located within 500 kb of an MR instrument more often than expected within the lipid-annotated candidate pool. The analysis was repeated at |log_2_FC| > 0.75 and > 1.0 to assess threshold sensitivity (**Supplementary Table 15**); GSEA was not repeated, as it ranks the full transcriptome without a fold-change threshold.

### 2.4 Machine learning-based signature construction and evaluation

All 143 combinations of 13 feature-selection methods and 11 classifiers were applied to the 459 candidates under ten-fold cross-validation; 13 glmBoost-based combinations returned constant predictions and were excluded, leaving 130 valid models. In the full-training analysis, LASSO logistic regression (24) at lambda.1se yielded one 12-gene candidate panel (**Supplementary Table 7**). Because exact selection depended on cross-validation partitioning and penalization, this set was treated as one candidate realization of the pipeline rather than a uniquely stable panel. Discrimination was quantified by the area under the receiver operating characteristic curve (AUC) (25). The final penalized model was evaluated by apparent training-set discrimination, independent external discrimination, external calibration, and decision-curve analysis (26). Febrile controls served as the reference category. The frozen penalized coefficients and complete prediction rule are reported in **Supplementary Tables 7**, **9**.

To quantify optimism from information leakage, the entire supervised pipeline—differential expression, intersection with the GeneCards list, and LASSO—was repeated under nested cross-validation with five independent ten-fold partitions (50 outer folds; **Supplementary Table 14**). The 143-combination benchmark was not repeated within each outer fold; nested cross-validation evaluated the prespecified DEG–GeneCards–LASSO pipeline only. For external validation, the penalized cv.glmnet model fitted in GSE73461 at lambda.1se was frozen and applied directly to GSE68004 with the 459 predictors supplied in the training-set order. No unpenalized post-LASSO refitting, outcome-dependent external standardization, coefficient re-estimation, or threshold re-estimation was performed. The classification threshold was selected from training-set predictions and fixed before external evaluation. Full-precision non-zero coefficients, the intercept, and the fixed threshold are provided in **Supplementary Table 9**. Selected analysis scripts, intermediate R data objects, and the GeneCards query output are deposited at https://doi.org/10.5281/zenodo.22080371.

### 2.5 Immune infiltration landscape analysis

Relative proportions of 22 immune cell types were estimated using CIBERSORT with the LM22 reference matrix (27). Samples failing the permutation-based significance filter (1,000 iterations, P < 0.05) were excluded. Spearman correlations between each marker and the estimated immune-cell fractions were calculated. Because complete blood counts were unavailable, the CIBERSORT estimates were compared with complementary marker-gene scores derived from the same bulk-expression matrix. The marker panels comprised canonical lineage-restricted genes that were not included in the LM22 reference matrix, and mature and immature granulocyte markers were assessed separately (**Supplementary Table 17**). This within-dataset analysis assessed computational concordance and reduced direct feature overlap with LM22, but it did not provide independent biological validation.

### 2.6 Exploratory *in vitro* transcriptional assessment

#### 2.6.1 Cell culture and inflammatory model

Human umbilical vein endothelial cells (HUVEC-SV40) were purchased from Procell Life Science & Technology (Wuhan, China). Cells were maintained in Endothelial Cell Medium (10% FBS, 1% penicillin-streptomycin) at 37 °C under 5% CO_2_. For inflammatory stimulation, cells seeded in 6-well plates were treated with recombinant human TNF-α at 0, 10, 20, or 50 ng/mL for 24 h (28).

#### 2.6.2 Quantitative Real-Time PCR (qRT-PCR)

Total RNA was isolated using TRIzol (Invitrogen, USA) and reverse-transcribed to cDNA. qRT-PCR was performed with SYBR Green on a CFX96 Touch system (Bio-Rad, USA), targeting CETP, KREMEN1, CCL23, VCAM-1, ICAM-1, and IL-8. Expression was normalized to GAPDH and quantified by the 2−ΔΔC^t^ method. Primer sequences are listed in **Supplementary Table 2**.

### 2.7 Statistical analysis

Bioinformatic analyses were performed in R (version 4.2.1); *in vitro* data were analyzed with GraphPad Prism (version 9.0). Continuous variables are expressed as mean ± SD or median (interquartile range). Two-group comparisons used Student’s t test or the Wilcoxon rank-sum test; multigroup comparisons used one-way ANOVA followed by Dunnett’s *post hoc* test. Two-sided P values or FDR-adjusted P values <0.05 were considered statistically significant.

### 2.8 Ethical statement

This study used publicly available, pre-anonymized GWAS summary statistics and GEO transcriptomic datasets collected under informed consent by the original investigators. Additional ethical approval was not required.

## 3 Results

### 3.1 MR screening of candidate exposures for association with KD susceptibility

Two-sample MR was applied to 422 candidate exposures—419 lipid/metabolic traits and three immune-cell phenotypes—against the FinnGen KD GWAS (**Figures 2A, B**; **Supplementary Table 4**). Nine traits reached nominal significance (P < 0.05), fewer than the approximately 21 expected by chance, and none survived Bonferroni correction or false-discovery-rate control (minimum Benjamini–Hochberg q = 0.979). All nine nominal hits were evaluated in ebi-a-GCST90014243 using a uniform follow-up procedure (**Supplementary Table 18**). Eight of the nine point estimates were directionally concordant with the FinnGen estimates, but none reached an unadjusted P < 0.05 in the second cohort (minimum P = 0.0928); consequently, none met either the descriptive nominal-concordance criterion or a multiplicity-adjusted replication criterion. For L_VLDL_FC_pct, the FinnGen IVW estimate was OR = 0.276 (95% CI 0.081–0.941, P = 0.040), whereas the second-cohort estimate was OR = 0.586 (95% CI 0.148-2.324, P = 0.447; 21 harmonized instruments). The additional sensitivity analyses of L_VLDL_FC_pct are therefore presented as *post hoc* exploratory analyses of a VLDL-composition trait with a comparatively large instrument set, not as evidence that it was the strongest association or that it replicated. For the FinnGen estimate, 50 independent instruments were extracted and 42 survived harmonization (F-statistics 30.3–2,337.6; median 50.8), mapping to canonical lipid loci including PCSK9, APOB, LPA, LPL, ABCA1, LDLR, and APOE (**Supplementary Tables 3**, **13**).

**Figure 2:**
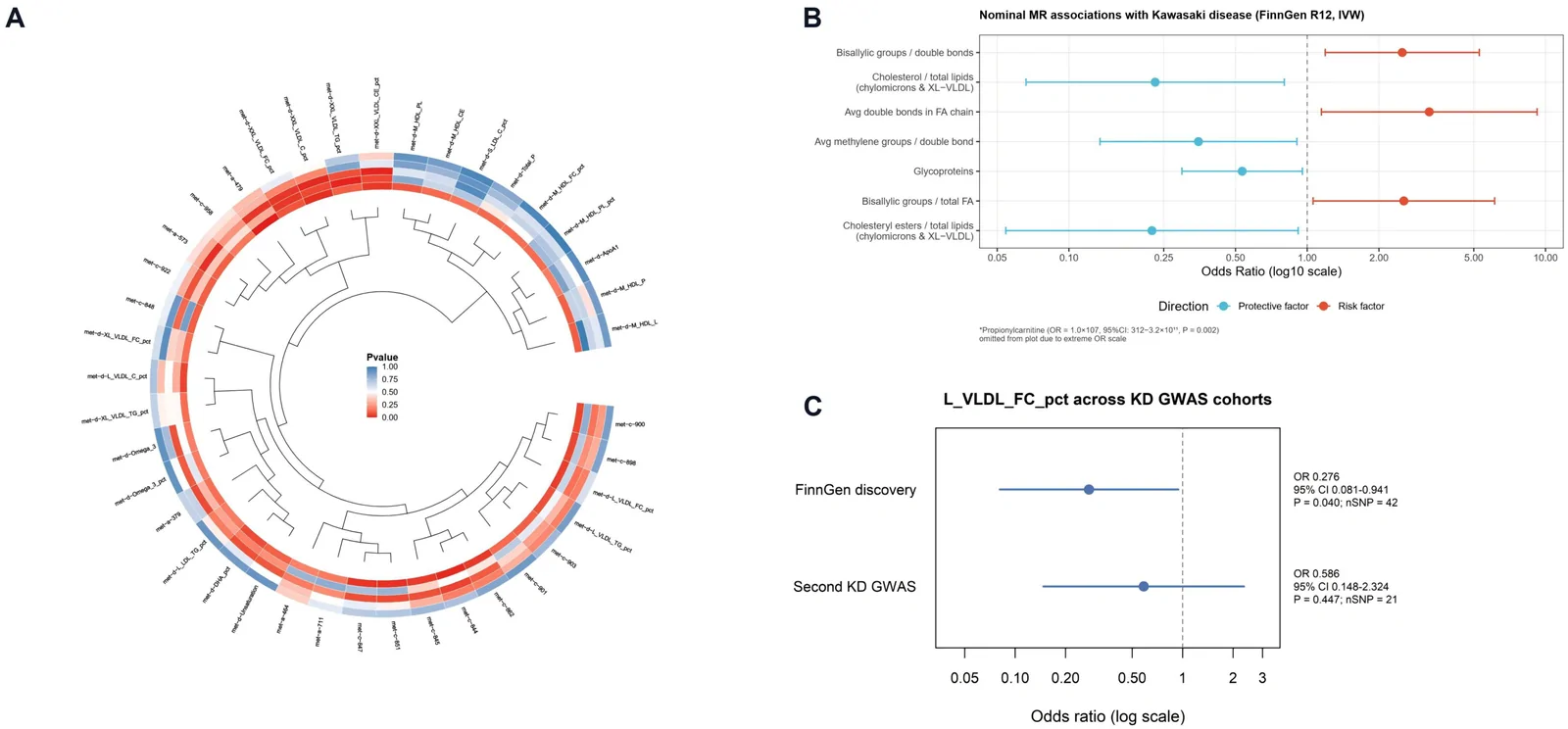
MR associations between candidate exposures and Kawasaki disease susceptibility. **(A)** Circular heatmap illustrating the associations of the 422 screened exposures with KD; complete results are provided in **Supplementary Table 4**. **(B)** Forest plot showing MR estimates for seven nominally significant exposure traits in FinnGen. L_VLDL_FC_pct is shown separately in panel C, whereas propionylcarnitine was omitted because its extreme OR scale would obscure the remaining estimates. Blue denotes OR < 1 and red denotes OR > 1. **(C)** L_VLDL_FC_pct estimates in FinnGen (OR = 0.276, 95% CI 0.081–0.941, P = 0.040; 42 instruments) and the second KD GWAS (OR = 0.586, 95% CI 0.148-2.324, P = 0.447; 21 instruments). The point estimates were below 1 in both cohorts, but the second-cohort interval included the null and did not provide nominal or multiplicity-adjusted evidence of replication. Complete second-cohort results for all nine FinnGen nominal hits are provided in **Supplementary Table 18**.

Sensitivity analyses (**Supplementary Table 12**) detected no heterogeneity (Cochran’s Q P = 0.675) and no outliers by MR-PRESSO (global P = 0.676). The MR-Egger intercept was not significant but was borderline (intercept = −0.110, P = 0.091) and should not be read as a clean negative. Leave-one-locus-out analysis across 34 independent loci retained the protective direction throughout (OR range 0.177–0.343), although only 24 of the 34 analyses retained P < 0.05. In the five pairwise multivariable MR models, the ORs were 0.175–0.546; estimates were farther from 1 after adjustment for apolipoprotein B or LDL cholesterol and closer to 1 after adjustment for HDL cholesterol or apolipoprotein A1; the full six-exposure model was not identified (conditional F-statistics 0.23–0.27). Scatter, funnel, single-SNP forest, and leave-one-out plots appear in **Supplementary Figure 1**.

Reverse MR was uninformative. No locus reached genome-wide significance; at a relaxed threshold of P < 5 × 10^−6^, the 43 sub-threshold variants had effect-allele frequencies of 7.8 × 10^−5^ to 6.7 × 10^−3^ and mapped to lncRNAs or pseudogenes, and only two could be harmonized, yielding a null estimate (IVW β = −0.0014, P = 0.900; **Supplementary Table 12**). Neither direction provided evidence of an effect of VLDL composition on KD susceptibility that survived multiple-testing correction.

### 3.2 Identification of KD-specific transcriptomic signatures against febrile controls

The GSE73461 analytic training cohort (77 KD and 146 FC; **Supplementary Table 1**) was used as the discovery cohort after exclusion of one KD sample with entirely missing expression values. Principal component analysis showed a partial transcriptomic shift between KD and the bacterial and viral febrile-control subgroups (**Supplementary Figure 2**). Limma identified 1,358 DEGs (|log_2_FC| > 0.585, adjusted P < 0.05; **Figures 3A, B**).

**Figure 3:**
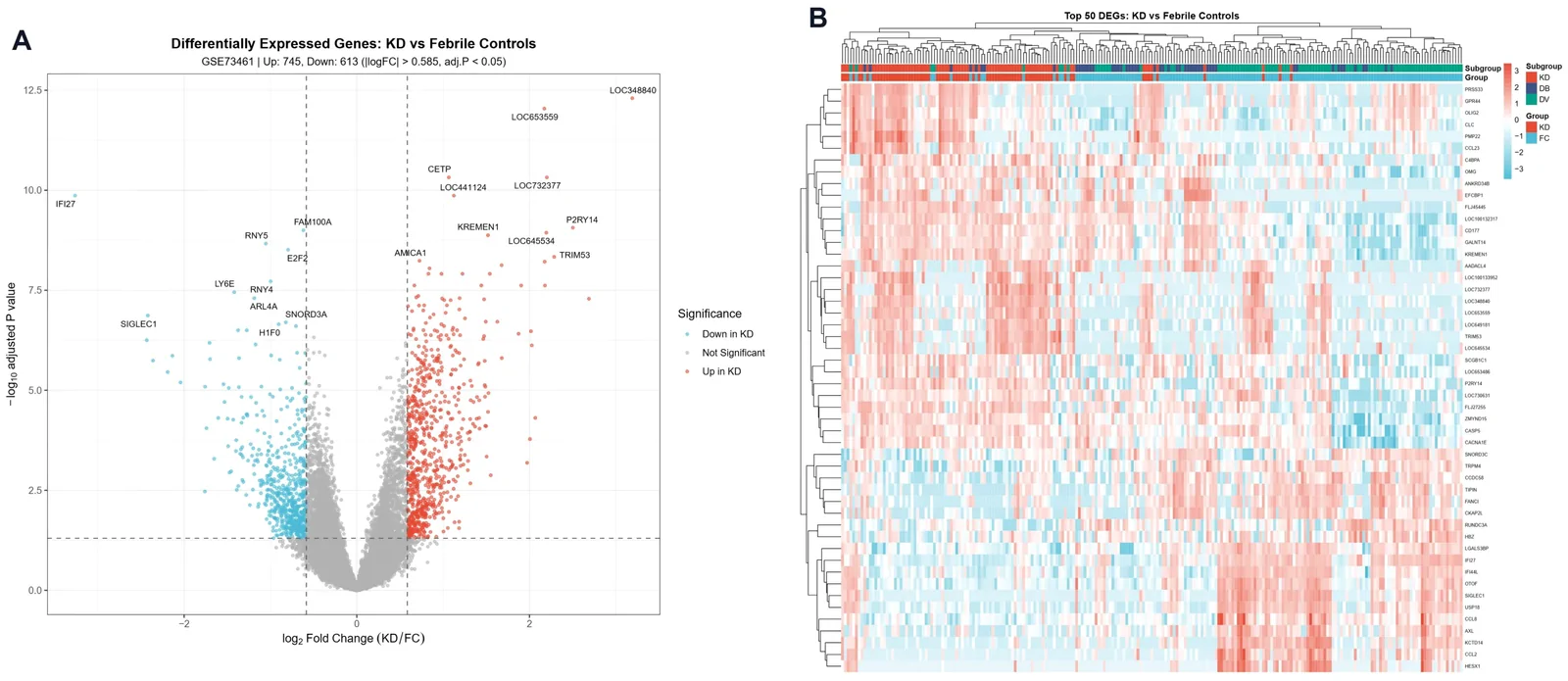
Differential gene expression between KD and febrile controls in the training cohort. **(A)** Volcano plot of DEGs (|log_2_FC| > 0.585, adjusted *P* < 0.05). **(B)** Hierarchical clustering heatmap of the top 50 DEGs.

To define a lipid-related candidate set, the 1,358 DEGs were overlaid with 9,603 lipid-metabolism-related genes curated from GeneCards, yielding 459 candidates (**Figure 4A**; **Supplementary Table 5**). GO Biological Process enrichment was dominated by leukocyte migration and chemotaxis, and KEGG analysis highlighted NET formation among the most enriched pathways (**Figures 4B, C**). Transcriptome-wide GSEA indicated enrichment of a NET formation-related gene set in KD (**Figure 4D**); over-representation analysis identified NET formation at the primary threshold but not at the stricter thresholds, which produced smaller gene lists (**Supplementary Table 16**). The threshold-independent GSEA result was therefore used to describe transcriptome-wide pathway enrichment.

**Figure 4:**
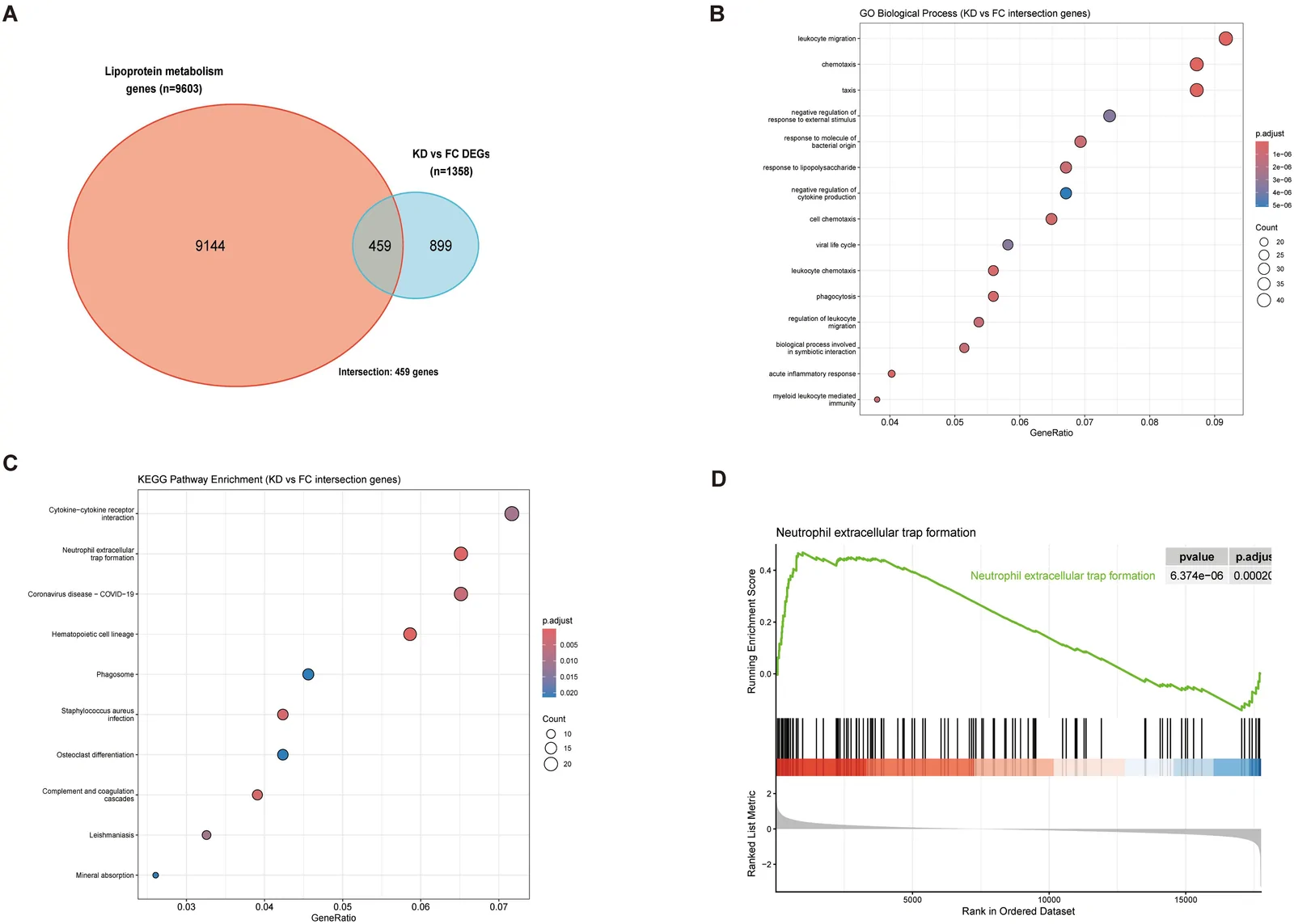
Functional enrichment of lipid-related candidate genes. **(A)** Venn diagram: intersection of 1,358 KD-specific DEGs with 9,603 lipid metabolism–related genes, yielding 459 candidates. **(B)** GO Biological Process enrichment highlighting leukocyte migration and chemotaxis. **(C)** KEGG pathway enrichment, including neutrophil extracellular trap (NET) formation. **(D)** GSEA plot showing a global transcriptomic shift toward NET formation in KD.

Two of the twelve signature genes lie within 500 kb of one of the 42 L_VLDL_FC_pct instruments: CETP (31.4 kb from rs75911530) and MT1G (containing rs904770). Permutation testing (10,000 draws of 12 genes from the 444 candidates with mappable coordinates; null mean 0.67, empirical P = 0.141; **Supplementary Figure 3**) showed that this overlap does not exceed chance expectation.

Threshold-specific reruns at |log_2_FC| thresholds of >0.585, >0.75, and >1.0 yielded panels containing 19, 11, and 12 genes, respectively (**Supplementary Table 15**). Panel size does not decrease monotonically with a stricter threshold because the candidate pool, cross-validation partition, and selected penalty all change together across reruns. These reruns assess feature-selection sensitivity only. Because they used different cross-validation partitions and produced different selected sets, their discrimination is not comparable with that of the frozen primary penalized model and is not reported. Nine of the original twelve genes were retained across all three reruns. The 19-gene result obtained in an independent rerun at the primary threshold further shows that exact panel composition depends on cross-validation partitioning and penalization.

### 3.3 Machine learning-based construction and independent evaluation of the 12-gene candidate panel

In the exploratory benchmark, Enet (α = 0.7) + Ridge achieved the highest cross-validated AUC (0.982) but retained 69 genes (**Supplementary Table 6**). To obtain a compact panel suitable for subsequent presentation and assay development, we used LASSO at lambda.1se, which selected 12 genes and yielded a non-nested cross-validated AUC of 0.970. CETP and KREMEN1 were the strongest single-gene discriminators (**Figure 5C**).

**Figure 5:**
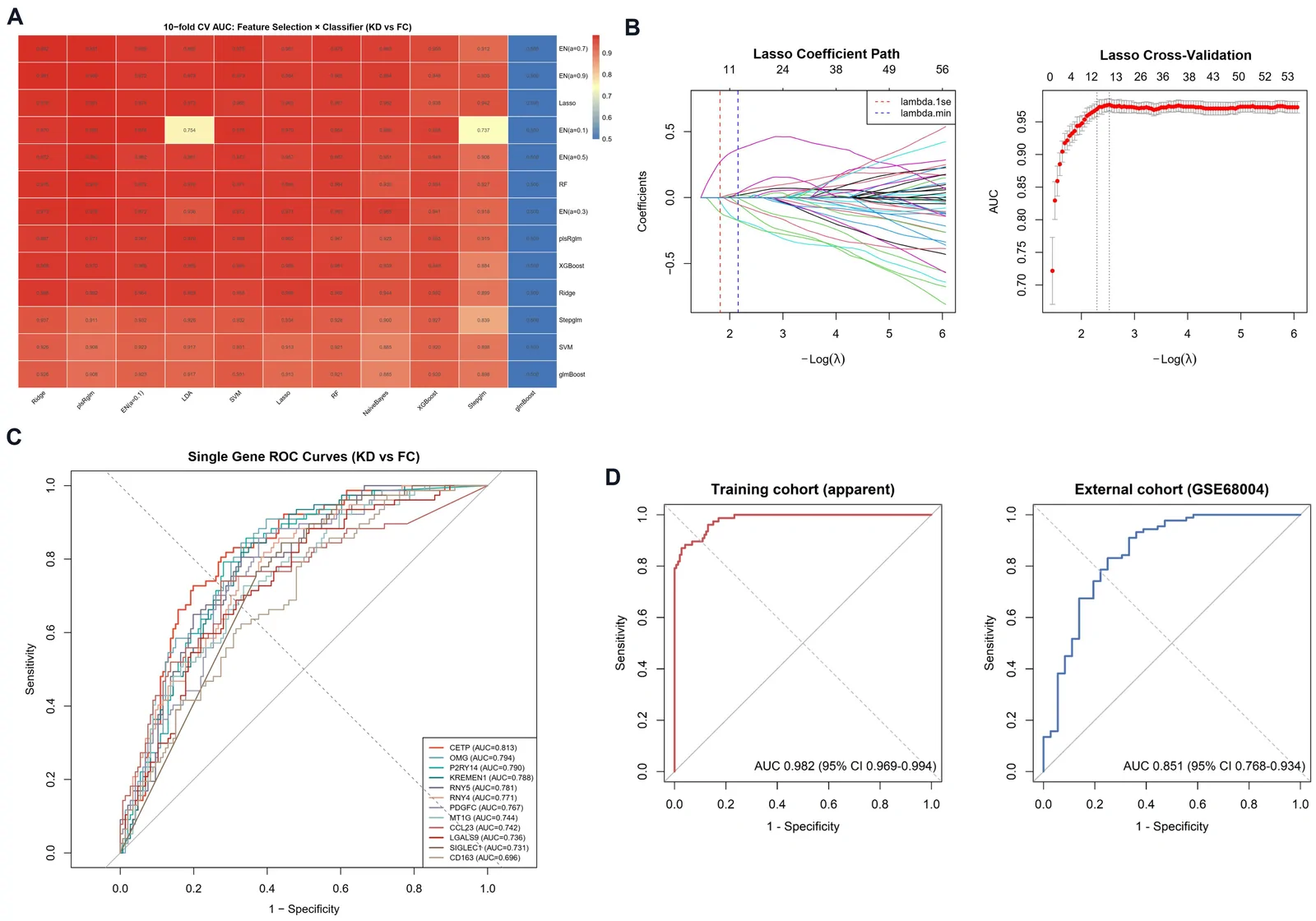
Construction and external validation of the candidate transcriptomic model. **(A)** Heatmap of cross-validated AUC across 130 valid algorithm combinations (**Supplementary Table 6**). **(B)** LASSO coefficient profile and cross-validation curve showing the full-training fit that yielded one 12-gene candidate panel at lambda.1se. **(C)** Single-gene ROC curves. **(D)** Frozen penalized LASSO model: training cohort (left, apparent AUC = 0.982, 95% CI 0.969-0.994) and independent validation GSE68004 (right, AUC = 0.851, 95% CI 0.768–0.934). The saved penalized model was applied without refitting or cross-cohort batch correction.

Nested cross-validation of the prespecified DEG-GeneCards-penalized-LASSO pipeline yielded an AUC of 0.957 (SD 0.006; **Supplementary Table 14**, **Supplementary Figure 4**). The final penalized model fitted to all GSE73461 samples achieved an apparent AUC of 0.982 (95% CI 0.969-0.994) and, when applied unchanged to GSE68004, an external AUC of 0.851 (95% CI 0.768–0.934; **Figure 5D**). At the fixed training-derived threshold of 0.441, sensitivity was 0.831 (95% CI 0.740-0.895), specificity 0.722 (95% CI 0.560-0.842), PPV 0.881, NPV 0.634, and accuracy 0.800 (74 TP, 15 FN, 26 TN, and 10 FP; **Supplementary Table 7**). External calibration remained imperfect: the calibration intercept was 0.957, the calibration slope was 2.071, the Brier score was 0.175, and the mean predicted probability was 52.5% versus an observed KD prevalence of 71.2% (**Figure 6**). Recalibration and prospective evaluation are required before clinical use.

**Figure 6:**
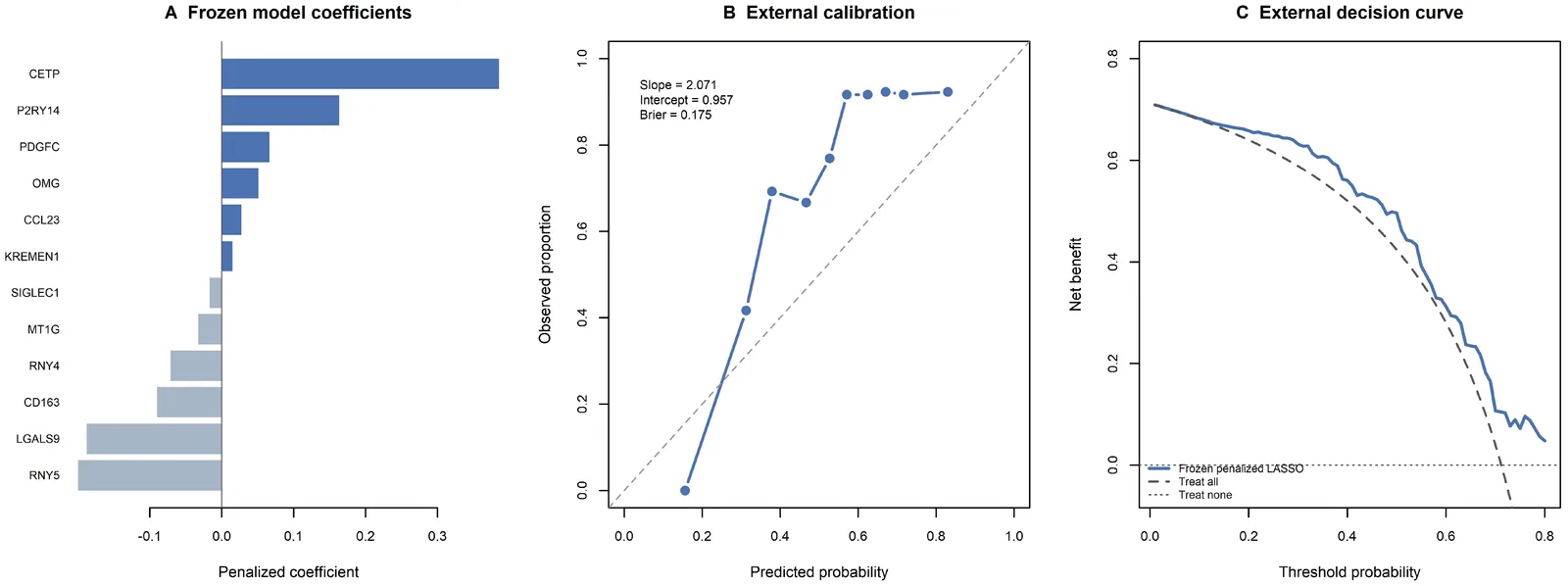
Frozen penalized model and external utility assessment. **(A)** Non-zero coefficients of the penalized LASSO model fitted in GSE73461 at lambda.1se. **(B)** External calibration in GSE68004; the diagonal denotes perfect calibration. Calibration intercept = 0.957, slope = 2.071, and Brier score = 0.175. **(C)** External decision-curve analysis for the frozen penalized model compared with treat-all and treat-none strategies. The calibration and decision-curve results are exploratory and require prospective validation.

Under nested cross-validation, ten of the twelve genes were re-selected in ≥88% of 50 outer folds, including six selected in every fold (**Supplementary Table 14**). SIGLEC1 and KREMEN1 were selected in 74% and 10% of folds, respectively. KREMEN1 was correlated with OMG (ρ = 0.63), PDGFC (ρ = 0.61), and CETP (ρ = 0.56), consistent with instability under L1 penalization in the presence of correlated predictors. KREMEN1 was therefore not a stable multivariable feature. Its separate biological interest rests on marginal KD-versus-FC upregulation (log_2_FC = 1.519, adjusted P = 1.33 × 10^−9^), a univariate AUC of 0.788, the largest acute-to-convalescent decrease (FDR = 5.6 × 10^−2^), and dose-dependent endothelial induction. The 12-gene set should be interpreted as one candidate realization of the DEG–GeneCards–LASSO pipeline. The saved full-training fit and full-precision coefficients define a reproducible fixed prediction rule, but rerunning feature selection under a different cross-validation partition is not expected to recover an identical gene set.

### 3.4 Frozen penalized model and external utility assessment

The final frozen model comprised 12 non-zero gene coefficients at lambda.1se (**Figure 6A**; **Supplementary Tables 7**, **9**). External calibration and decision-curve analyses are shown in **Figures 6B, C**. The calibration results indicate that the model should not be interpreted as a calibrated probability tool in its current form. Decision-curve results are exploratory because they were obtained in a retrospective cohort with 89 KD cases and 36 febrile controls and require confirmation in a prospectively sampled clinical population.

### 3.5 Dynamic changes in the signature genes between acute and convalescent phases

In GSE63881, 170 KD patients with paired acute-phase and convalescent-phase samples were analyzed (one of 171 patients lacked a convalescent sample). The majority of the 12 signature genes shifted toward convalescent levels (**Figure 7**). KREMEN1 showed the largest acute-to-convalescent decrease (FDR = 5.6 × 10^−2^), followed by CETP (FDR = 1.4 × 10^−11^) and CCL23 (FDR = 1.6 × 10^−6^). All three were elevated during the acute phase and were lower in convalescent-phase samples, consistent with an association with the acute-to-convalescent sampling phase and meriting prospective evaluation as acute-to-convalescent phase markers.

**Figure 7:**
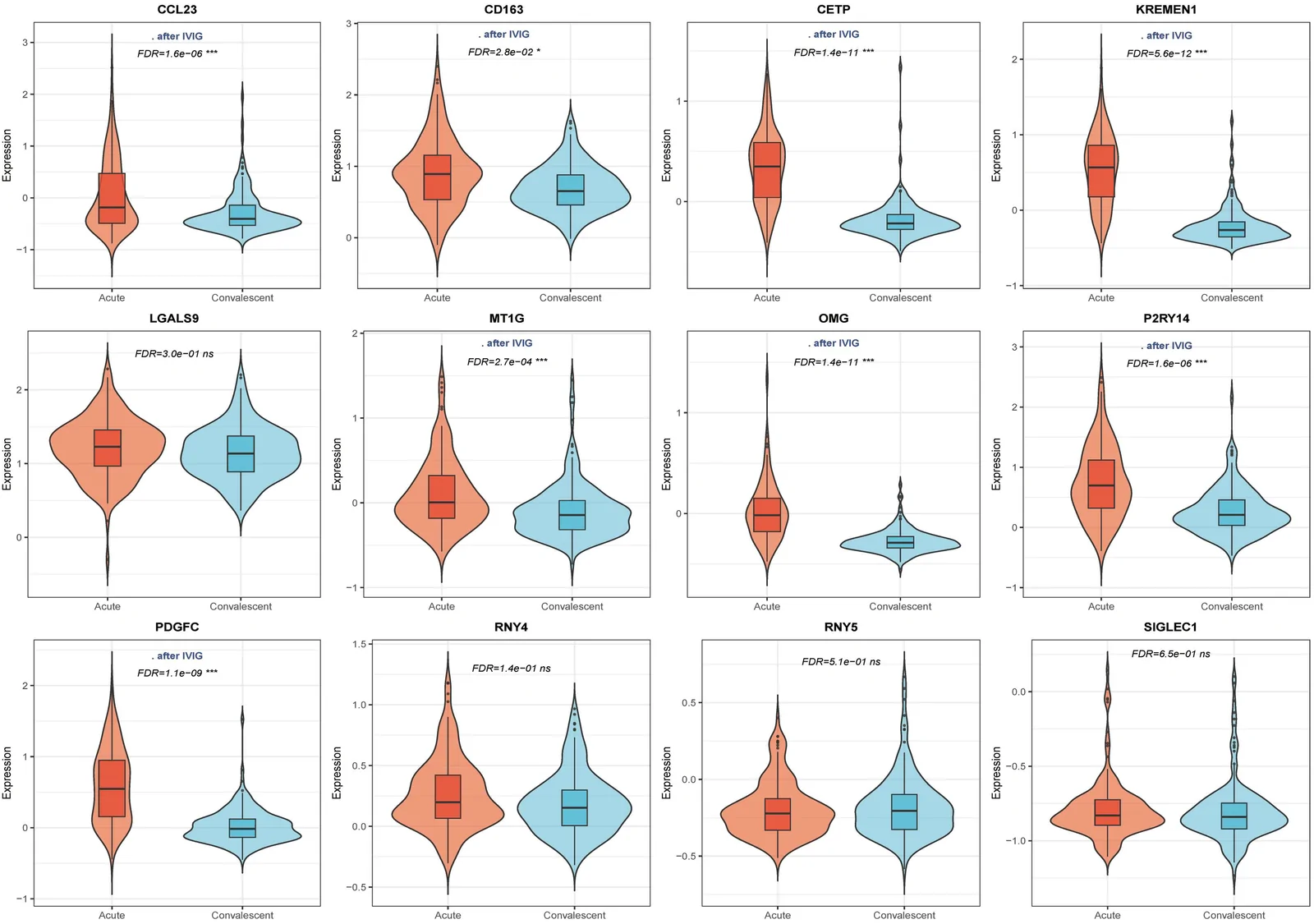
Dynamic expression changes of the signature genes between acute and convalescent phases. Violin plots of matched acute-phase and convalescent-phase expression in GSE63881 (n = 170 paired patients with KD). CETP (FDR = 1.4 × 10^−11^), KREMEN1 (FDR = 5.6 × 10^−2^), and CCL23 (FDR = 1.6 × 10^−6^) showed the largest acute-to-convalescent decreases. Paired Wilcoxon signed-rank tests with Benjamini–Hochberg correction. Benjamini–Hochberg-adjusted significance of the paired Wilcoxon signed-rank test: ns, FDR ≥ 0.05; *FDR < 0.05; **FDR < 0.01; ***FDR < 0.001.

### 3.6 Immune microenvironment landscape and gene–immune associations

CIBERSORT deconvolution indicated higher estimated neutrophil and monocyte fractions in KD than in febrile controls, together with lower estimated fractions of CD8^+^ T cells, resting memory CD4^+^ T cells, and resting NK cells (**Figures 8A, B**; **Supplementary Table 8**). In a complementary within-dataset analysis, the CIBERSORT neutrophil estimate correlated with the mature-neutrophil marker score (ρ = 0.741, P = 4.5 × 10^−40^), and the marker score was higher in KD than in febrile controls (P = 1.8 × 10^−7^; **Supplementary Table 17**, **Supplementary Figure 5**). The CD8^+^ T-cell marker score was also lower in KD (P = 0.0025). The monocyte marker score was directionally higher in KD, but the group difference was not statistically significant. Mature and immature granulocyte marker scores correlated with the CIBERSORT neutrophil estimate in opposite directions (ρ = 0.741 and −0.133, respectively), indicating that the positive concordance was driven by the mature-neutrophil marker set. Because both approaches used the same expression matrix, these results provide computational concordance rather than independent validation of immune-cell abundance.

**Figure 8:**
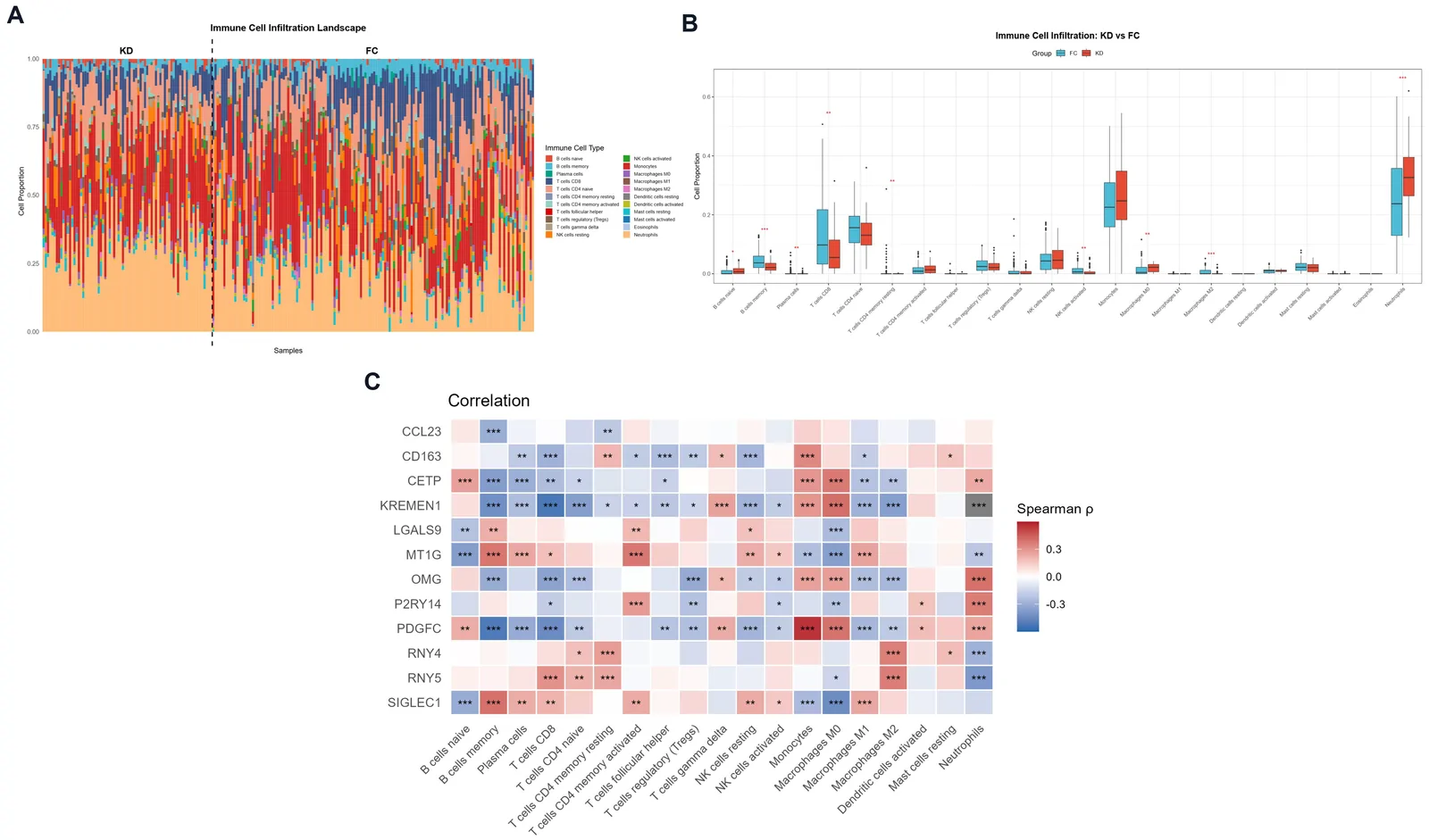
Immune infiltration landscape and gene–immune correlation analysis. **(A)** Stacked bar plot of 22 immune cell types in KD and febrile controls estimated using CIBERSORT. **(B)** Box plots comparing estimated immune-cell proportions; detailed statistical results are provided in **Supplementary Table 8**. **(C)** Gene–immune correlation heatmap based on unadjusted Spearman correlations. Asterisks indicate unadjusted P values (*P < 0.05, **P < 0.01, and ***P < 0.001). After Benjamini–Hochberg correction across the full gene–immune correlation matrix, only the association between KREMEN1 and the CIBERSORT-estimated neutrophil fraction remained significant.

After Benjamini–Hochberg correction across the full gene–immune correlation matrix, KREMEN1 was the only signature gene whose association with the CIBERSORT-estimated neutrophil fraction remained significant (ρ = 0.653; **Figure 8C**). This association was also observed with the mature-neutrophil marker score (ρ = 0.779). CETP showed nominal correlations with the CIBERSORT neutrophil and monocyte estimates, but these did not survive multiple-testing correction; it showed moderate correlations with the mature-neutrophil marker score (ρ = 0.400) and monocyte marker score (ρ = 0.382). CCL23 correlations were weak under both approaches; its relevance to the inflammatory process rests on its acute-to-convalescent expression change and endothelial transcriptional induction rather than on immune-cell association.

### 3.7 Exploratory transcriptional response of selected markers in an endothelial inflammatory model

TNF-α treatment increased VCAM-1 and ICAM-1 expression in a dose-dependent manner (both P < 0.01 at 20 ng/mL; **Figure 9A**). CETP and KREMEN1 expression also increased (**Figure 9B**). CCL23 increased with TNF-α concentration and was significant at 20 and 50 ng/mL, whereas IL-8 increased at all tested concentrations (**Figure 9C**). These are transcriptional responses to a shared inflammatory stimulus and were not tested for mediation.

**Figure 9:**
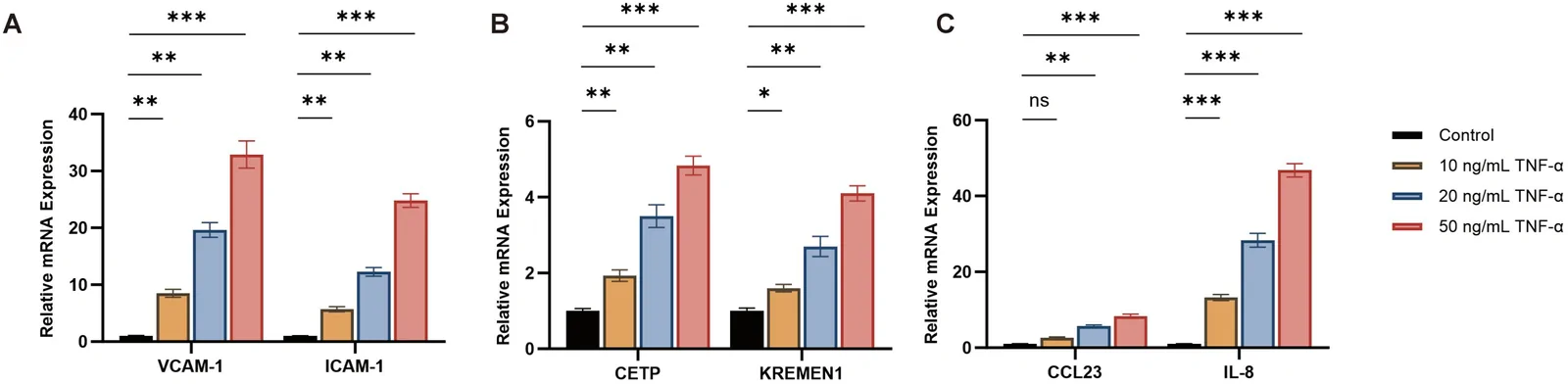
*In vitro* expression of selected markers in TNF-α-stimulated HUVECs. **(A)** Dose-dependent upregulation of VCAM-1 and ICAM-1 following 24-h stimulation with TNF-α (0–50 ng/mL). **(B)** Induction of CETP and KREMEN1 in activated endothelial cells. **(C)** CCL23 increased with TNF-α concentration and was significant at 20 and 50 ng/mL, whereas IL-8 increased at all tested TNF-α concentrations. These mRNA-level findings were obtained in venous endothelial cells with three biological replicates and no protein-level confirmation; they should therefore be considered hypothesis-generating. One-way ANOVA with Dunnett’s test; mean ± SD, n = 3 per group. ns, not significant; *P < 0.05, **P < 0.01, ***P < 0.001 versus control.

## 4 Discussion

Evaluating the classifier against febrile infection rather than health yields a discrimination estimate that corresponds to the decision clinicians actually face. Separately, KREMEN1 emerged as a candidate for biological follow-up on marginal, longitudinal, and immune-associated grounds, despite its low selection frequency in the multivariable pipeline.

A persistent weakness in the KD biomarker literature is the choice of comparator. Classifiers trained against healthy children routinely exceed AUC 0.95 (5) but may primarily capture a broad inflammatory signal rather than KD-specific biology, and performance decreases when the comparator group includes bacterial or viral infections (1, 5). Our training cohort used exclusively definite infections as controls, and external validation was performed without cross-cohort batch correction in GSE68004, which included adenovirus and group A Streptococcus cases. Nested cross-validation of the prespecified penalized pipeline yielded an AUC of 0.957, whereas the final frozen penalized model achieved an external AUC of 0.851. This reduction, together with imperfect external calibration, indicates that the model remains a candidate classifier rather than a clinically deployable diagnostic tool. Recalibration and prospective evaluation in a pediatric emergency population are required.

None of the nine discovery-stage nominal hits reached an unadjusted P < 0.05 in the second KD GWAS, although eight point estimates were directionally concordant. For L_VLDL_FC_pct, the second-cohort estimate was imprecise and included the null (OR = 0.586, 95% CI 0.148-2.324, P = 0.447). Its detailed sensitivity analyses should therefore be viewed as *post hoc* exploration of a VLDL-composition trait rather than evidence of replication. More broadly, the MR screen remained hypothesis-generating and did not define or narrow the transcriptomic candidate set or establish genetic mediation of the selected genes. Consistent with this separation, permutation testing showed that the proximity of signature genes to MR instruments did not exceed chance expectation within the lipid-annotated candidate pool.

The CIBERSORT-derived immune profile was directionally consistent with the neutrophil predominance reported in acute KD vasculitis (11, 13) and with evidence that coronary arteritis can develop in RAG1-deficient mice lacking adaptive immunity (29). KREMEN1 had a small coefficient in the frozen multivariable model and was selected in only 10% of outer folds; it is therefore not presented as a stable or essential component of the diagnostic panel. Its separate biological interest arises from marginal KD-versus-FC upregulation, univariate discrimination, the largest acute-to-convalescent decline among the candidate genes, and its within-dataset association with neutrophil-related estimates. These findings support further evaluation of KREMEN1 as a candidate immune-associated marker but do not establish immune-cell specificity, recruitment, or mechanism. The weaker, correction-sensitive associations observed for CETP and CCL23 should be interpreted cautiously. Although CETP catalyzes cholesteryl ester exchange between HDL and apolipoprotein B-containing lipoproteins (30), the present data do not establish that CETP, CCL23, or KREMEN1 regulates immune-cell recruitment in KD.

The HUVEC experiments show that CETP, KREMEN1, and CCL23 can be co-induced with canonical endothelial activation and chemotaxis markers by a shared inflammatory stimulus; this does not establish a mechanistic link between lipid-metabolic dysregulation and chemokine production at the endothelial surface (31). Whether these genes act upstream of chemokine release, or merely respond to the same upstream signal, will require loss-of-function experiments.

KREMEN1, which encodes a receptor involved in Wnt/β-catenin signaling (32), showed the steepest acute-to-convalescent decline. If this association is confirmed prospectively, serial measurement of KREMEN1 and the other phase-associated markers could be evaluated as a complement to conventional assessment of disease phase.

Several limitations should be considered. The MR screen was exploratory: no trait survived Bonferroni or false-discovery-rate correction across 422 exposures, and the systematic second-cohort analysis of the nine discovery-stage nominal hits was *post hoc*. None of the nine follow-up estimates reached an unadjusted P < 0.05; the L_VLDL_FC_pct estimate was directionally concordant but imprecise and included the null, and its additional sensitivity analyses were *post hoc* and do not establish replication. Power was a limiting factor throughout: the two outcome GWAS contributed 79 and 119 KD cases respectively, so all MR estimates carry wide confidence intervals, and a non-significant second-cohort result does not distinguish absence of effect from insufficient power. The transcriptomic candidate pool was generated independently of the MR analysis, and no colocalization, SMR/HEIDI, or transcriptome-wide association analysis linked L_VLDL_FC_pct to the selected genes. All instruments derive from European-ancestry GWAS, whereas KD is most prevalent in East Asian populations (33) with potentially different susceptibility architecture; ancestry-matched replication would require a substantially larger East Asian KD GWAS together with a matched metabolomic GWAS, neither currently available to us. A retrospective East Asian pediatric cohort with paired lipid profiles and complete blood counts would be the most informative next step.

The entire pipeline was built on publicly deposited retrospective cohorts; prospective evaluation in a pediatric emergency population is needed to establish diagnostic thresholds. The independent validation cohort contains only 36 febrile controls against 89 KD cases, encompassing adenovirus and group A Streptococcus infections but not measles, drug hypersensitivity, systemic juvenile idiopathic arthritis, or MIS-C. Consequently, specificity is estimated from only 36 controls, and the corresponding NPV is likewise subject to substantial uncertainty. Moreover, the 71.2% KD prevalence in GSE68004 limits the transferability of PPV and NPV to unselected febrile populations (**Supplementary Table 7**).

The *in vitro* component quantified mRNA only, used TNF-α in venous rather than coronary arterial endothelial cells, and included no protein-level confirmation or loss-of-function experiments. The specific next steps are protein-level validation by Western blot and ELISA, siRNA knockdown of the core markers with adhesion-molecule and chemotaxis readouts, replication in coronary artery endothelial cells, and stimulation with acute-phase KD serum or the LCWE murine model (34).

The reverse-direction MR was uninformative, not negative, so an effect of KD liability on VLDL composition cannot be excluded. Exact panel composition was sensitive to cross-validation partitioning and penalization; accordingly, the frozen 12-gene fit is reproducible as a prediction rule, but the selected gene set should not be interpreted as uniquely stable, and a future implementation should verify panel composition in an independent derivation cohort.

## 5 Conclusions

A frozen penalized transcriptomic model showed moderate discrimination between KD and rigorously classified febrile controls in an independent cohort. The 12-gene set is one realization of a partition-sensitive selection pipeline, not a uniquely stable panel. The MR analysis remained exploratory and separate from transcriptomic candidate definition; no screened exposure survived multiplicity correction, and none of the nine nominal discovery-stage hits reached an unadjusted P < 0.05 in the second KD GWAS. KREMEN1 retains separate biological interest on the basis of marginal, longitudinal, and within-dataset immune-association findings, but not as a stable multivariable feature. Prospective validation, recalibration, and functional studies are required before clinical or mechanistic interpretation.

## Data Availability

The datasets presented in this study can be found in online repositories. The names of the repository/repositories and accession number(s) can be found in the article/**Supplementary Material**.
