# Supplementary material for: A lipid-related transcriptomic signature for distinguishing Kawasaki disease from febrile infections: a Mendelian randomization and machine-learning study
Source: Front Immunol. 2026 Sep 30;17:1851332. doi: 10.3389/fimmu.2026.1851332 (PMC13661412; doi:10.3389/fimmu.2026.1851332)
Supplement: Supplementary file 1 [file Supplementaryfile1.docx]

**Supplementary Materials**

**Supplementary Figure S1.**  Sensitivity analyses for the MR association of L_VLDL_FC_pct with Kawasaki disease susceptibility. For each plot type, the left panel shows the primary FinnGen cohort and the right panel shows the second KD GWAS used for post hoc follow-up (ebi-a-GCST90014243). Scatter plots, funnel plots, single-SNP forest plots, and leave-one-out analyses are shown for both cohorts.

**Supplementary Figure S2.** Principal component analysis (PCA) of the training cohort (GSE73461). (A) PCA plot showing global transcriptomic distribution of KD patients (n = 77) compared to febrile controls (FC, n = 146). (B) Subgroup PCA showing the distributions of KD, definite bacterial (DB), and definite viral (DV) infections.

**Supplementary Figure S3.** Permutation test of overlap between the 12 genes included in the frozen penalized model and the 42 Mendelian randomization instruments. Gray bars show the null distribution from 10,000 random draws of 12 genes from the 444-gene candidate pool; the red line marks the observed value of 2 (CETP and MT1G). The observed overlap does not exceed chance expectation (empirical P = 0.141).

**Supplementary Figure S4.** Receiver operating characteristic curves for the prespecified DEG–GeneCards–penalized-LASSO pipeline under nested cross-validation and for the non-nested LASSO procedure at lambda.1se. The red curve represents the pointwise mean of five ROC curves generated from the out-of-fold predictions of five repeated ten-fold nested cross-validation runs. The gray curve represents the fold-size-weighted mean of ten ROC curves from the non-nested ten-fold procedure. The corresponding mean AUCs were 0.957 and 0.970, yielding an optimism estimate of 0.013. The non-nested lambda.min estimate of 0.977 is reported separately in Supplementary Table S14 and was not used to quantify optimism. The dashed diagonal indicates chance discrimination.

**Supplementary Figure S5.** Within-dataset concordance between the CIBERSORT-estimated neutrophil fraction and a complementary mature-neutrophil marker-gene score derived from the same bulk-expression matrix. Each point represents one sample passing the CIBERSORT permutation filter and is colored by group. The marker score was calculated as the mean gene-wise z-score of FCGR3B, CSF3R, S100A12, MME, and SELL. The CIBERSORT-estimated neutrophil fraction correlated with the mature-neutrophil marker score (Spearman ρ = 0.741, P = 4.5 × 10⁻⁴⁰). The solid line and shaded band show the linear fit and its 95% confidence interval for visualization. Because both measures were derived from the same bulk-expression matrix, this analysis represents computational concordance and not independent biological validation.
